# Plasticity after cognitive training reflected in prefrontal local field potentials

**DOI:** 10.1016/j.isci.2022.104929

**Published:** 2022-08-12

**Authors:** Balbir Singh, Zhengyang Wang, Xue-Lian Qi, Christos Constantinidis

**Affiliations:** 1 Department of Biomedical Engineering, Vanderbilt University, Nashville, TN 37235, USA; 2 Neuroscience Program, Vanderbilt University, Nashville, TN 37235, USA; 3 Department of Neurobiology & Anatomy, Wake Forest School of Medicine, Winston-Salem, NC 27157, USA; 4 Department of Ophthalmology and Visual Sciences, Vanderbilt University Medical Center, Nashville, TN 37232, USA

**Keywords:** Behavioral neuroscience, Systems neuroscience, Cognitive neuroscience

## Abstract

Learning to perform a new cognitive task induces plasticity of the prefrontal cortex generally involving activation of more neurons and increases in firing rate; however, its effects on single neurons are diverse and complex. We sought to understand how training affects global measures of neural activity by recording and analyzing local field potentials (LFPs) in monkeys before and after they learned to perform working memory tasks. LFP power after training was characterized by a reduction in power in 20–40 Hz during the stimulus presentations and delay periods of the task. Both evoked power, synchronized to task events, and induced power exhibited this decrease after training. The effect was consistent across tasks requiring memory of spatial location and stimulus shape. Error trials were characterized by a lack of LFP power ramping around the fixation onset. Our results reveal signatures of cortical plasticity in LFPs associated with learning to perform cognitive tasks.

## Highlights

• LFP power in the 20–40 Hz range decreases after training in a working memory task
• The decrease generalizes across spatial and shape working memory tasks
• Effects were most pronounced in the mid-dorsal prefrontal subdivision
• The effects did not reverse for error trials or passive presentation after training

## Graphical abstract

**Figure:**
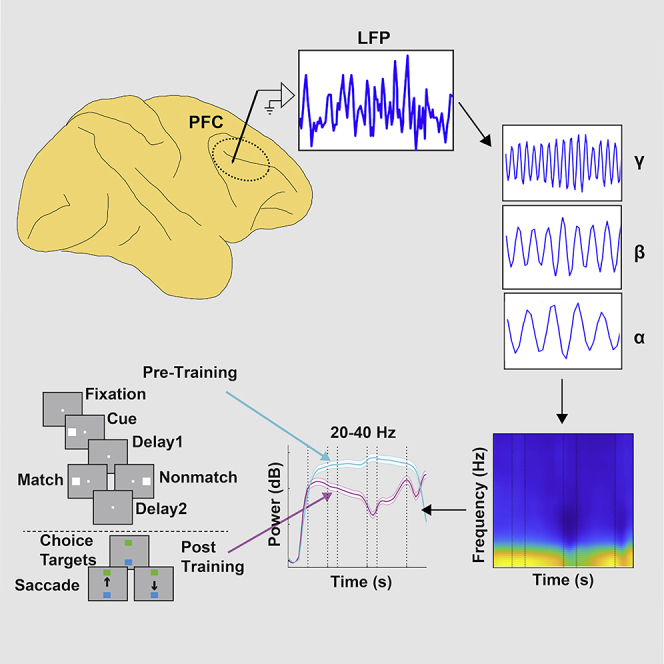

## Introduction

Training monkeys to perform a new working memory task results in long-lasting changes in neural activity, including increases in the number of neurons that are activated after appearance of stimuli and an overall increase in activity (Mendoza-Halliday and Martinez-Trujillo, 2017; Meyer et al., 2011). These changes are evident even when the trained monkeys are tested with passive presentation of stimuli in the same fashion they did prior to training (Riley et al., 2018). Moreover, greater mastery of the task, evidenced by improved performance, results in greater changes in activity that reflect the task performance levels at each point in time (Qi et al., 2011; Tang et al., 2019). The effects depend greatly on the cortical area being sampled; gradients of plasticity have been identified across the anterior-posterior and dorsoventral axes with greater plasticity observed in more anterior and ventral areas (Constantinidis and Qi, 2018; Meyer et al., 2011; Riley et al., 2018). These effects are not uniform, however, even within a single subdivision, with the majority of neurons often not responding to any task conditions, and a great diversity of responses observed at early as well as late stages of training (Tang et al., 2022). We were motivated therefore to investigate global effects of training by examining the power of the prefrontal local field potentials (LFP).

The LFP represents summation of ionic currents in a limited cortical volume, in the order of 0.1–0.2 mm radius (Buzsaki, 2004; Kajikawa and Schroeder, 2011). During presentation of stimuli, correlated bottom-up inputs can serve to synchronize population neuronal spiking, and phases of synchronized excitation by pyramidal neurons followed by inhibition by interneurons can thus produce rhythmicity in the field potentials (Fries, 2009). LFP rhythmicity in the gamma frequency range, in particular, is well known to emerge in the delay period of working memory tasks and to be tuned for information held in memory, e.g. spatial location (Holmes et al., 2018; Pesaran et al., 2002) or other features of a remembered visual stimulus (Lundqvist et al., 2016; Sakamoto et al., 2022; Tanigawa et al., 2022; Wutz et al., 2018). Gamma frequency rhythmicity has also been associated with other types of functions likely to be engaged during performance of a cognitive task, such as bottom-up attention (Buschman and Miller, 2007). Gamma rhythms are not the only frequency range modulated by cognitive factors; beta frequency oscillations have been shown to be indicative of top-down attention and decision-making (Bastos et al., 2015; Haegens et al., 2011; van Kerkoerle et al., 2014). Beta and gamma oscillations are readily detectible in other extracellular field recordings (such as EEG or MEG) and are thus a reliable marker of underlying cognitive processes impacting neural circuit interactions (Roux and Uhlhaas, 2014; Uhlhaas and Singer, 2011) and cognitive functions including working memory and top-down control (Helfrich and Knight, 2016; Siegel et al., 2012).

Learning to perform a new cognitive task would be expected to influence functions such as working memory, attention, and cognitive control, and therefore, we wished to identify the effects of training on LFP rhythmicity. Our current study investigated systematically the impact of training to perform working memory tasks on LFP potential measures of rhythmic firing in other subdivisions of the prefrontal cortex, in training involving spatial and object working memory.

## Results

**Figure 1:**
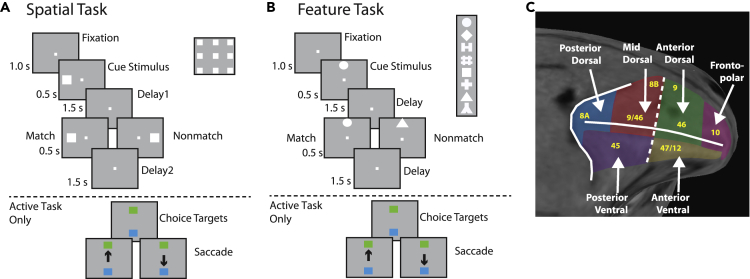
Recording area and task (A) Sequence of frames indicates events in the spatial match-to-sample task. The animals were required to maintain center fixation throughout both active and passive task trials. At the end of active tasks trials however, monkeys were required to make a saccade to a green target if the stimuli matched or to a blue target if the stimuli did not match. (B) Shape feature match-to-sample task, 8 possible shapes in a session shown in the inset. (C) Anatomical location of areas where recordings were made in the lateral prefrontal cortex.

We recorded LFP activity from the lateral prefrontal cortex of two monkeys before and after they were trained to perform a match-non-match working memory task (Figures 1A and 1B). Two stimulus sets were used in these experiments, one varying the spatial location of a white square (spatial set), and one involving different geometric shapes (feature set). The monkeys had to observe two stimuli presented in sequence, and after a delay period to determine whether they were the same or not. They indicated their judgment by making an eye movement to a blue or green choice target. Prior to training, the exact same stimuli sets were presented to the same animals, under the same timing, which at this point only viewed them passively and were rewarded solely for maintain fixation. Choice targets never appeared prior to training. The animals were exposed to the two stimulus sets over multiple sessions prior to the onset of recording in the pre-training phase and were fully familiar with them during data acquisition.

Sufficient LFP data before and after training were obtained from three prefrontal subdivisions, the posterior-dorsal, mid-dorsal, and anterior-dorsal areas (Riley et al., 2017). A total of 5163 trials from 34 electrodes of LFP recordings obtained with the spatial task of Figure 1A were available in the posterior-dorsal area prior to training and 8062 trials from 69 electrodes after training. Similarly, 15351 trials from 96 electrodes were available from the mid-dorsal area before and 17831 trials from 139 electrodes after training. Additionally, 11408 trials from 88 electrodes were available from the posterior-ventral area before and 18587 trials from 152 electrodes after training.

### Decrease of 20–40 Hz LFP power after training

**Figure 2:**
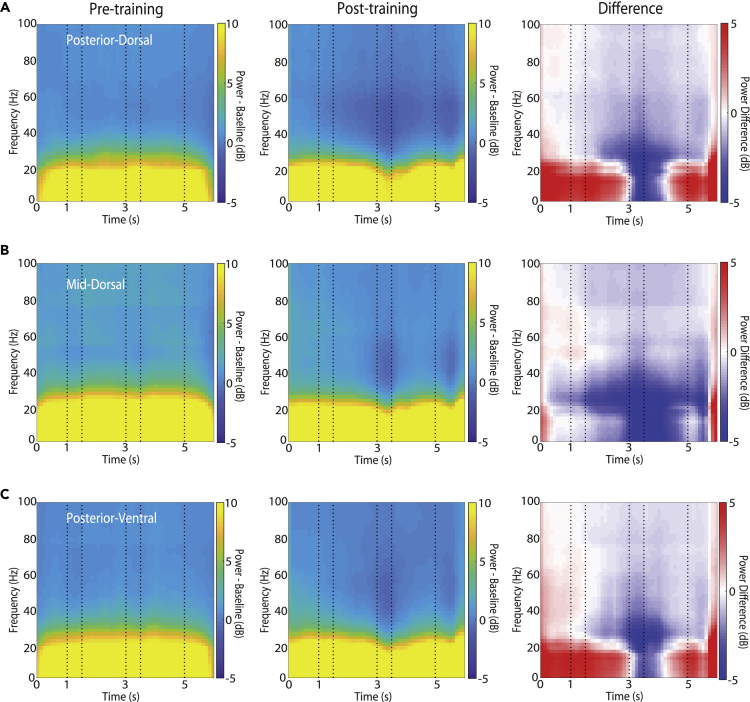
Induced LFP power for spatial stimuli LFP spectral power recorded with the spatial stimulus set from the prefrontal cortex, prior to training (left column) and after training (middle column), as well as their difference (right column). Power is plotted as a function of time, after subtracting the mean power computed in the inter-trial interval at each frequency band. Horizontal lines indicate time of the two stimulus presentations (at 1–1.5 and 3–3.5 s) and choice target presentation (at 5 s). Results are shown separately for (A) posterior-dorsal (n = 5163 and 8062 trials for pre-training and post-training recordings, respectively), (B) mid-dorsal (n = 15351 and 17831) and (C) posterior-ventral areas (n = 11408 and 18587).

**Figure 3:**
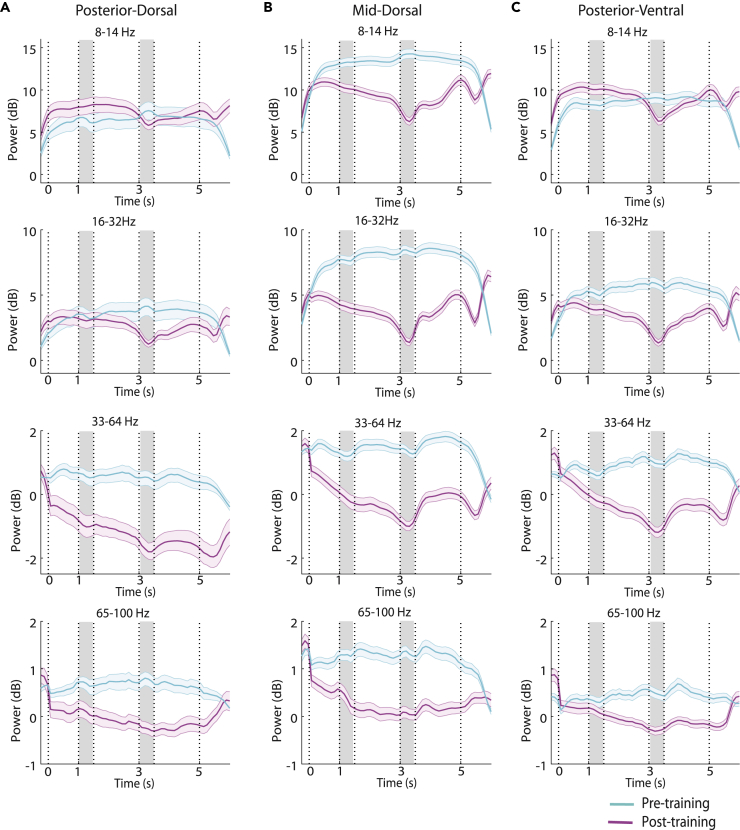
Time course of power in 4 frequency bands in 3 prefrontal areas Time course of induced spectral power in the spatial task, after subtracting the mean computed in the inter-trial interval at each frequency band. Data are shown in the alpha (8–14), beta (16–32), gamma (33–64), and high-gamma (65–100) frequency band, comparing pre-training and post-training results. Gray bars indicate time of the two stimulus presentations (at 1–1.5 and 3–3.5 s); dotted lines represent onset of fixation point (at 0s) and choice target presentation (at 5 s). Results are shown separately for (A) posterior-dorsal (n = 34 and 69 sites for pre-training and post-training, respectively), (B) mid-dorsal (n = 96 and 139), and (C) posterior-ventral areas (n = 88 and 152). Data are represented as mean (solid line) and its 5 and 95 percentiles estimated by subsampling 75% of the data 1000 times (shaded area).

We first examined how training to perform a working memory task altered the time course of induced LFP power, i.e. power computed independently at each trial and then averaged, and not necessarily synchronized at specific task events across trials. For each frequency, we first calculated the mean power value across the intertrial interval. We then subtracted this value (in logarithmic units) from the power time series to obtain spectrograms of mean-corrected power changes (Figure 2). We then examined differences between training stages, areas, and conditions centering around task events. The time course of power computed in four different frequency bands is shown in Figure 3. Data analyzed in this fashion allowed us to determine spectral power changes across time and how these differed between conditions. Across all three prefrontal areas, the most consistent effect of training on average spectral power was a decrease in power in lower frequencies (<40 Hz, Figure 2, right column) after the end of the cue presentation period. The interval where this decrease was most evident was the second stimulus presentation (Figure 2, center column); some power decrease was also evident in the first and second delay periods. Prior to training, the second stimulus appearance had no specific meaning, but after training this is the critical time interval that required a categorical judgment by the subject.

To test how robust power changes around task events were across electrode sites, we calculated power in five discrete task epochs (fixation period, cue presentation, first delay, sample presentation, and second delay), then averaged power computed in this interval for all trials obtained from each electrode and treated it as a single observation. We relied on a general linear model analysis, comparing this power values using a 3-way ANOVA, with factors pre- or post-training condition, prefrontal subdivision, and task epoch. This analysis was performed separately (i.e. a separate 3-way ANOVA model was computed) for power in each of four different frequency bands: alpha, defined as 8–14 Hz, beta (16–32 Hz), gamma (33–64 Hz), and high gamma (65–100 Hz).

The most salient effect of training was a decrease in power, centered in the 20–40 Hz range in terms of frequency, and temporally, at the time of the second stimulus presentation. A main effect of training was significant for all frequency bands, as training tended to have a strong overall decrease of power (*F*_*1,2860*_ = 14.1, p = 0.0002 for alpha; *F*_*1,*__*2860*_ = 200.02, p = 6.05E-44 for beta; *F*_*1,*__*2860*_ = 323.14, p = 1.54E-68 for gamma; *F*_*1,*__*2860*_ = 183.63, p = 1.4E-40 for high gamma). An interaction term of task epoch x training was significant for each frequency band, suggesting differential effects of training at different task epochs (*F*_*4,*__*2860*_ = 8.45, p = 8.9E-7 for alpha power, *F*_*4,*__*2860*_ = 12.19, p = 7.9E-10 at beta; *F*_*4,*__*2860*_ = 6.68, p = 2.4E-05 at lower gamma; *F*_*4,*_ _*2860*_ = 4.64, p = 0.001 at high gamma). Most importantly, spectral power in the fixation period generally increased after training, especially in the alpha band (Figure 2, right column, interval 0–1 s).

Different prefrontal subdivisions showed differential plasticity of LFP rhythmicity across frequency ranges, after training. The mid-dorsal region had the most profound effect of training, showing significant power decreases across all frequency bands in the two delays and the second stimulus presentation (3-way ANOVA and Tukey post-hoc test, evaluated at the α = 0.05 significance level). In contrast, the effect of training on the posterior-ventral region was restricted to the beta and gamma bands, showing significant power decrease in the two delay periods and the second stimulus presentation period while no significant power changes were detected in the alpha band. The training had a lesser effect on the lower frequencies for the posterior-dorsal region, where no significant power changes were found in the alpha and beta frequency bands. The power decrease was only significant during the second stimulus presentation in the gamma frequency bands.

An important caveat in comparing LFP recordings of the two training phases was that more cumulative electrode penetrations had been performed in the prefrontal cortex in the post-training phase than in the pre-training one. We wished to examine therefore whether potential tissue damage from repeated penetrations could account for the differences in power that we examined. We therefore conducted recordings in the second hemisphere of one animal after training, where no prior penetrations had been performed prior to training. The results are shown in Figure S1. This analysis confirmed a decrease in alpha and beta power after training in both the resampled, left hemisphere (Figure S1A), and the newly sampled right hemisphere (Figure S1B).

**Figure 4:**
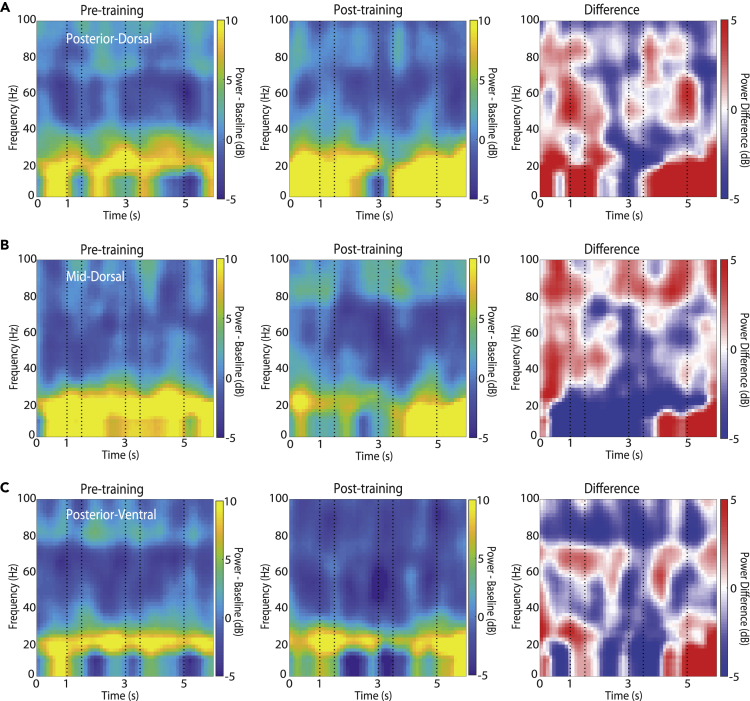
Evoked LFP power for spatial stimuli LFP spectral power recorded with the spatial stimulus set from the prefrontal cortex during the time course of the trial, prior to training (left column) and after training (middle column), as well as their difference (right column). Power is plotted as a function of time, after subtracting the mean power of the inter-trial interval at each frequency band. Conventions are the same as in Figure 2. Results are shown separately for (A) posterior-dorsal (n = 5163 and 8062 trials for pre-training and post-training recordings, respectively), (B) mid-dorsal (n = 15351 and 17831), and (C) posterior-ventral areas (n = 11408 and 18587).

We also examined evoked LFP power, i.e. power computed after averaging LFP signals of a site across trials first, which tends to emphasize power synchronized at specific task events across trials. We thus computed LFPs synchronized to the onset of the cue and then determining power of this averaged signal. The duration of stimulus presentations and delay periods were fixed in our task, and thus LFP activity could be well aligned to task events, which could be anticipated (Figure 4). The findings were generally in line with the results of the induced LFP power: the predominant effect of training was a decrease in power, most prominent in the 20–40 Hz frequency range. The time course of evoked power was generally more complex however, with increases and decreases synchronized at different time events. We relied on a general linear model analysis, using the same 3-way ANOVA model again, with factors pre- or post-training condition, prefrontal subdivision, and task epoch. This analysis also showed a significant effect of training at all frequency bands (*F*_*1,2860*_ = 106.1, p = 1.9E-24 at alpha; *F*_*1,2860*_ = 253.69, p = 8.6E-55 at beta; *F*_*1,2860*_ = 161.72, p = 4.4E-36 at gamma and *F*_*1,2860*_ = 96.26, p = 2.3E-22 at high gamma). As was the case for induced power, changes in evoked power after training were also epoch dependent, as evidenced by a significant interaction between training phase and epoch factors (*F*_*4,*__*2860*_ = 13.69, p = 4.6E-11 for alpha power, *F*_*4,*_ _*2860*_ = 12.15, p = 8.5E-10 at beta; *F*_*4,*__*2860*_ = 4.9, p = 0.0006 at lower gamma; *F*_*4,*__*2860*_ = 2.98, p = 0.018 at high gamma).

### Consistent LFP power changes after training across tasks

The results presented so far were based on analysis of the spatial stimulus set (Figure 1A). We wished to test whether training in the other variant of the working memory task (the feature task of Figure 1B) produced similar changes in the induced LFP. Stimuli in this set differed in terms of their shape rather than their spatial location. Additionally, all sessions analyzed here involved presentation of the stimuli in the foveal location, where neuronal selectivity for shapes is strongest (Meyer et al., 2011; Riley et al., 2018). A total of 1757, 5492, and 12907 trials of LFP recordings collected from 15, 47, and 105 electrodes were available from the posterior-dorsal, mid-dorsal, and posterior-ventral prefrontal cortex, prior to training, respectively. An additional 3702, 6403, and 13528 trials were available from 37, 57, and 121 electrodes, respectively, post training.

**Figure 5:**
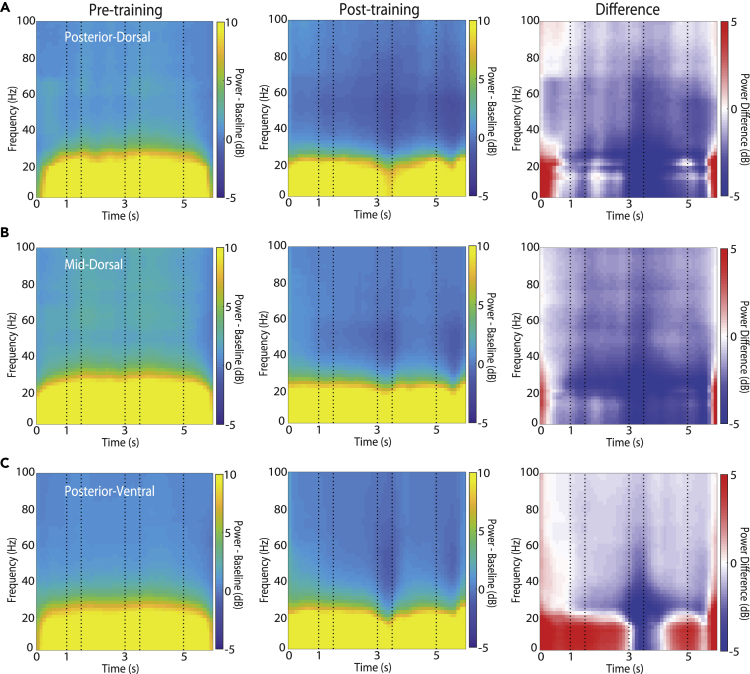
Induced LFP power spectrum for feature stimuli LFP spectral power recorded with the feature stimulus set from the prefrontal cortex during the time course of the trial, prior to training (left column) and after training (middle column), as well as their difference (right column). Conventions are the same as in Figure 2. Results are shown separately for (A) posterior-dorsal (n = 1757 and 3702 trials for pre-training and post-training, respectively), (B) mid-dorsal (n = 5492 and 6403), and (C) posterior-ventral areas (n = 12907 and 13528).

A decrease in induced power in lower frequencies after training was evident in this case, too (Figure 5). The time course of power in each frequency band we defined is shown in Figure S2. We relied on the same general linear model analysis using a 3-way ANOVA, with factors pre- or post-training condition, prefrontal subdivision, and task epoch. This analysis confirmed a significant effect of training in all frequency bands (*F*_*1,1880*_ = 17.38, p = 3.2E-05 at alpha; *F*_*1,1880*_ = 151.66, p = 1.43E-33 at beta; *F*_*1,1880*_ = 323.1, p = 8.6E-67 at lower gamma; *F*_*1,1880*_ = 255.6, p = 4.9E-54 at high gamma). In this case, too, changes in power after training were epoch dependent, as evidenced by a significant interaction between training phase and epoch factors *F*_*4,*__*1880*_ = 6.41, p = 4.03E-05 for alpha power, *F*_*4,*__*1880*_ = 9.67, p = 9.7E-08 for beta; *F*_*4,*__*1880*_ = 5.59, p = 0.0002 for gamma; *F*_*4,*__*1880*_ = 5.31, p = 0.0003 for high gamma). This overall decrease in alpha, beta, and gamma power especially in the second stimulus period for the feature task was also confirmed in recordings from the same hemisphere before and after training (Figure S3A), as well as recordings from a previously non-sampled hemisphere after training (Figure S3B).

The feature working memory task similarly revealed effects of training that differed systematically between areas. Overall, the mid-dorsal and posterior-ventral regions showed higher plasticity in the feature task. Comparing power in individual epochs, a significant decrease in beta, low gamma, and high gamma power for the mid-dorsal area and posterior-ventral (Figures 5B and 5C) was evident at the first delay period, the second stimulus period, and second delay periods (3-way ANOVA and Tukey post-hoc test, p < 0.05 in each case). A decrease in alpha power was only significant during the second stimulus presentation for these two regions. On the other hand, a significant decrease in gamma power for posterior-dorsal area (Figure 5A) was only evident at the second stimulus period and second delay periods. No changes were significant for the posterior-dorsal region at any task epoch in the alpha and beta frequency ranges.

### Error trials and passive presentation reveal long-lasting effects of training

Changes in power after training do not necessarily imply that LFP spectral composition is associated with the performance of the trained task. We therefore wished to compare spectral power in groups of trials that resulted in correct and error performance, after training. We analyzed error trials that involved a saccade to the erroneous choice target (see Figures 1A and 1B), excluding from analysis trials that were aborted due to breaks in fixation that lead to premature termination of the trial. Our dataset in the spatial task consisted of 2821, 2318, and 3195 error trials, obtained from 65, 132, and 150 electrodes in the posterior-dorsal, mid-dorsal, and posterior-ventral regions, respectively (post-training). To compare conditions, we again used a 3-way ANOVA, with factors correct vs. error (rather than pre- or post-training condition), prefrontal subdivision, and task epoch.

**Figure 6:**
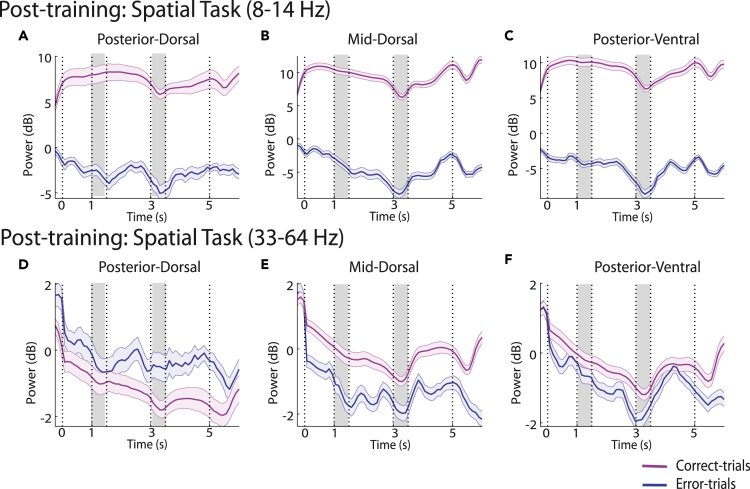
Error trials. Time course of spectral power in correct and error trials (A–C) Results are shown for the spatial stimulus set, in the alpha (8–14 Hz) frequency band. Conventions are the same as in Figure 3. (A) Time course of posterior-dorsal LFP power (n = 69 and 65 sites for correct and error trials, respectively). (B) Mid-dorsal power (n = 139 and 132). (C) Posterior-ventral power (n = 152 and 150). (D–F) Results for the gamma (33–64) frequency band.

We hypothesized that error trials would be characterized by higher spectral power during the time intervals of the two stimulus presentations and delay periods, resembling the pre-training phase. This did not turn out to be the case. Rather, broad-band power was lower in the fixation interval and remained so throughout the task epochs until the second delay period (Figure 6). The effect was highly consistent across all frequency bands (Figures 6A–6C). As a result, a significant main effect of correct vs. error trial type was present in the 3-way ANOVA (*F*_*1,3505*_ = 3368.18, p = 0 at alpha; *F*_*1,3505*_ = 1649.5, p = 6.92E-296 at beta; *F*_*1,3505*_ = 7.48, p = 0.006 at gamma; *F*_*1,3505*_ = 106.47, p = 1.3E-24 at high gamma). No significant interactions between correct/error status and task epoch were detected in any frequency band, as error-predicting LFP power appeared in the fixation period, while the subsequent within-trial temporal structure of LFP power remained largely intact.

The gamma frequency band (33–64 Hz) exhibited areal-specific differences between error and correct trials. The posterior-dorsal region alone showed significant higher LFP power in error trials (Tukey post-hoc test, evaluated at α = 0.05). This could be due to the posterior-dorsal being the only region to show decreased lower gamma power in the fixation period on correct trials (Figure S4). These results suggest that LFP power was diagnostic of performance: trials in which LFP power showed less ramping in the fixation period were more likely to result in errors.

**Figure 7:**
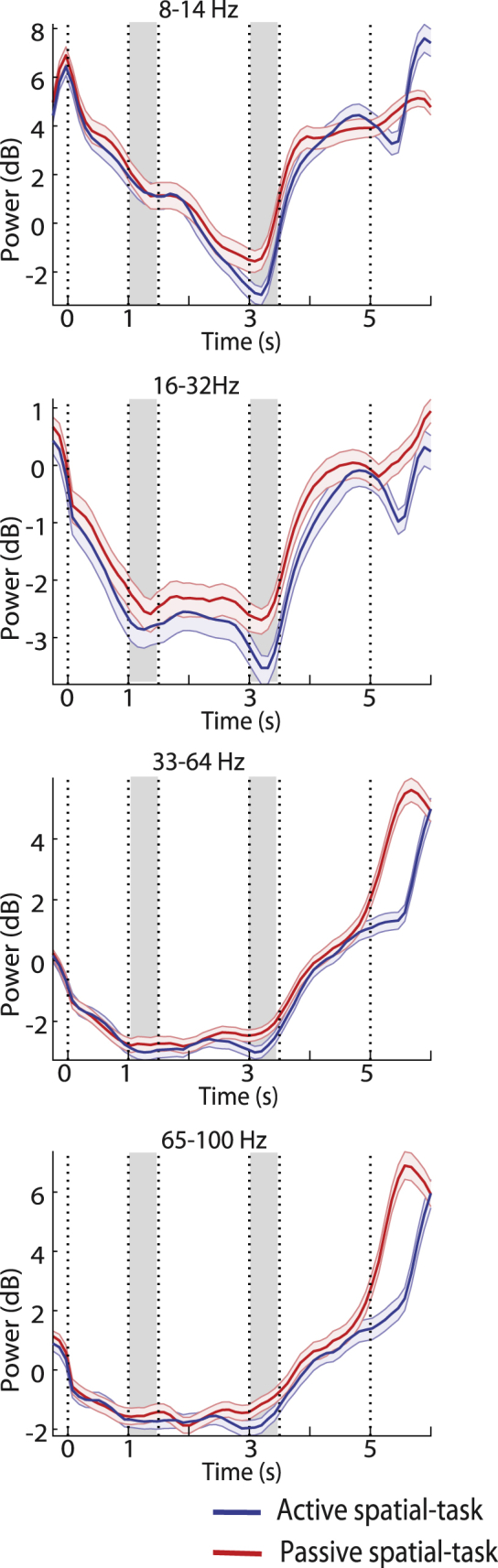
Passive presentation after training Time course of spectral power for trials performed after training, when the monkey performed the spatial task or viewed the same stimuli passively. Power is shown after subtracting the mean computed in the inter-trial interval at each frequency band. Means are shown for the alpha, beta, gamma, and high-gamma bands. Results from all available areas have been pooled together (n = 73 sites, in each case). Conventions are the same as in Figure 4.

As discussed earlier, recordings in the post-training phase differed with the pre-training phase in that the monkeys had both undergone training and executed a cognitive task rather than viewing the stimuli passively. To dissociate the effects of these two factors on spectral power, we obtained a set of recordings after training in which the monkeys passively viewed the stimuli, in a manner identical to pre-training. We then compared results from sites in which both active performance of the spatial task and passive presentation were available. Results from 7751 trials from 73 sites in the passive, and 7312 trials from the same sites in the active task, are shown in Figure 7. For this analysis, we pooled data from all available areas together and performed a 2-way ANOVA with factors passive-vs active presentation status, and epoch. The time course of LFP power evolution was surprisingly similar between the post-training active and passive tasks across all frequency bands. Only beta power was significantly yet slightly reduced for the active task (*F*_*1,725*_ = 7, p = 0.008) when averaged across epochs. Such decreases were not significant for any particular epoch examined post-hoc (Tukey post-hoc test, α = 0.05). The result suggested that learning to perform the task produced enduring changes in LFP spectral power in response to the same stimuli sequences, largely regardless of task conditions.

## Discussion

Our study analyzed LFP power in response to identical stimulus presentations before and after monkeys were trained to perform working memory tasks, which required them to maintain these stimuli in memory and make judgments about them. Neural oscillations have been implicated in a range of cognitive processes, including working memory and top-down control (Helfrich and Knight, 2016; Roux and Uhlhaas, 2014; Siegel et al., 2012; Uhlhaas and Singer, 2011), and we thus sought to determine to what extent observed patterns of LFP power in trained subjects are shaped by training and performing the task. We show that such training reorganizes LFP power, particularly at the 20-40 Hz range. A relative increase of alpha power was evident in the fixation period and a decrease during the presentation of the second stimulus and the delay periods of the task. Both induced and evoked spectral power showed this pattern. Such effects of training extended beyond a single task context, as evidenced by the highly consistent post-training temporal pattern of LFP power regardless of whether location (Figure 3) or shape (Figure S2) was to be remembered and whether overt reporting was required (Figure 7). Trials involving passive presentation of stimuli, after the monkeys had been trained in the task were characterized by higher overall power; however, the difference between active and passive tasks post-training was very subtle, compared to the pre-training stage. On the other hand, error trials involved further alpha power decrease, evident from the fixation period onward. These findings suggested that training produced lasting changes in the rhythmicity of LFP potentials, which did not reverse depending on task performance. Some specialization was evident between subdivisions of the prefrontal cortex in terms of both relative LFP power and effects of training. Contrary to expectations, power at the gamma frequency range, which has been implicated in the active maintenance of working memory (Holmes et al., 2018; Lundqvist et al., 2016; Pesaran et al., 2002; Tanigawa et al., 2022) was not increased in the trained phase of the experiment.

### Effects of training in neuronal responses

In a series of previous studies, involving a partially overlapping set of recordings used for LFP analysis here, we have documented that training in cognitive tasks produces a number of changes in the activity of prefrontal neurons (Qi and Constantinidis, 2013). These include the firing rate of neurons in response to task events, which generally increases after training by virtue of a greater proportion of prefrontal neurons responding to stimuli, and at higher mean firing rates, particularly in anterior and ventral prefrontal subdivisions (Meyer et al., 2011; Riley et al., 2018; Tang et al., 2022). An increase in firing rate was evident already during the fixation period and is consistent with the general increase in LFP power we observed now in the fixation interval. Training also produced more subtle changes in neuronal selectivity and representation of new task variables (Dang et al., 2021; Meyers et al., 2012), in agreement with other studies, as well, which have shown that learning of different tasks may not require global changes in prefrontal activity but may involve more modest changes in the selectivity of some neurons (Asaad et al., 1998; Sarma et al., 2016).

Training effects also involve changes in their trial-to-trial response variability and the correlation of firing rates between neurons, both of which typically decline (Qi and Constantinidis, 2012a; b). In other words, responses of individual neurons become less variable and more decoupled from nearby neurons. These effects are likely to have direct implications on coordinated measures of neural activity such as those captured by local field potentials and are consistent with the overall decrease in LFP power we observed after training. Task training also alters the time course and dynamics of firing rate, e.g. involving “ramping” of activity in the fixation interval, prior to the appearance of the cue (Kobak et al., 2016; Tang et al., 2022), and we in fact observed increased alpha power during the fixation interval, absence of which characterized error trials. The results we report here are likely to reflect the cumulative effects of such reorganization of neuronal activity and its dynamics.

Experimental results reported here were essentially captured at two “snapshots” in time, before training began and after it had been completed. In a separate series of experiments, we have recently reported a progressive decrease of frequency in the 20–45 Hz power in the LFPs recorded from the posterior-dorsal area of the prefrontal cortex as training progressed (Tang et al., 2022). Taken together, these studies suggest that training to perform the working memory task induces progressive decreases in power, evident both in evoked and induced LFP power, for both dorsal and ventral subdivisions of the prefrontal cortex, and during training in both spatial and feature working memory tasks.

### LFP power in different prefrontal subdivisions

The organization and functional role of the prefrontal cortex has been the matter of some debate (Constantinidis and Qi, 2018; Miller, 2000). Anatomical evidence suggests a relative segregation of spatial and feature information in the dorsal and ventral prefrontal cortex; however, this is more likely to be greater in posterior rather than anterior subdivisions of the prefrontal cortex (Constantinidis and Qi, 2018). Additionally, subdivisions along the anterior-posterior and dorsoventral axes of the PFC display varying degrees of plasticity after training (Li et al., 2020). Highest levels of plasticity after training have been documented in more anterior over posterior areas (Meyer et al., 2011; Riley et al., 2018) and for the ventral compared to the dorsal PFC (Meyer et al., 2011).

In agreement with other recent studies that have reported differential effects of spatial and non-spatial information on the spectral composition of LFPs in the ventral and dorsal prefrontal cortex (Sakamoto et al., 2022; Wutz et al., 2018), we observed subtle but distinct patterns of LFP power in the two subdivisions. We also found that training affects LFP power differentially as well, with the greatest changes in alpha and beta power after training observed for the mid-dorsal and posterior-ventral areas in the feature task, consistent with the anterior-posterior and dorsoventral axes of plasticity of firing rate changes (Riley et al., 2018).

### Changes in gamma power

LFP gamma power has been associated with the maintenance of information in working memory (Holmes et al., 2018; Lundqvist et al., 2016; Pesaran et al., 2002; Tanigawa et al., 2022). Specifically, stronger gamma-frequency LFP power is evident in sites where spiking activity is also elevated during working memory (Lundqvist et al., 2018; Wang et al., 2022). Because neuronal recordings from more sites after training exhibited persistent activity, and more stimulus-selectivity persistent activity was observed (Qi and Constantinidis, 2013), we tested whether an increase in gamma power in the delay intervals of the task was also evident. This was generally not the case; gamma power in the delay periods was unchanged or decreased for most conditions.

As mentioned, the trained monkeys had both learned and performed the new task. Bottom-up processing has been associated with increased gamma power (Bastos et al., 2015; van Kerkoerle et al., 2014) and in this sense, finding strong gamma power in the pre-training LFP is not surprising, considering that bottom-up factors dominated processing prior to learning to perform a cognitive task. It has been under-appreciated that gamma power may appear in LFP even before training, and therefore not only emerge during execution of working memory tasks, as in fact is the case for persistent firing rate (Meyer et al., 2007).

### Changes in other frequency bands

The most salient effect of training involved changes in power in the beta and alpha frequency bands. After training, broad-band power was decreased during the stimulus presentation and delay periods of the task. Beta power has been suggested as an inhibitory rhythm that maintains the status quo (Engel and Fries, 2010), and such a decrease in the stimulus and delay periods after training may be associated with a change in processing requirements in these epochs from the pre-training passive task when only fixation was required throughout all task periods. Alpha power in sensory cortex has been thought to reflect active suppression of task-irrelevant information (Clayton et al., 2015; Klimesch, 2012; Peylo et al., 2021). However, in the prefrontal cortex, the role of alpha power is less clear. It is known to be elevated during the stimulus presentation, but does not otherwise appear tuned to stimulus properties in the delay period (Holmes et al., 2018). Stimulation of the cholinergic forebrain tends to decrease alpha band rhythmicity which has been interpreted as stabilizing the activity of prefrontal neurons and making memory of the stimulus less likely to shift (Singh et al., 2022). In our experiment, decrease in alpha power tended to be centered on the appearance of the second stimulus, which after training acquired a unique context: the subject needed to perform a categorical judgment based on whether this constituted a match or nonmatch. Decrease in alpha power appears as a signature of this process.

### Limitations of the study

Our study had some limitations. Sufficient data were available from only three subdivisions of the prefrontal cortex. It is possible therefore that additional differences are present in areas not sampled and additional experiments will be required to address this question. Our analysis also relied exclusively on single-electrode measures of power, as recordings involved single electrodes, or arrays of electrodes tightly clustered within a single subdivision of the prefrontal cortex. Rhythmicity of neuronal activity is often evident in coherence between areas at specific frequency bands (Hagan and Pesaran, 2022; Salazar et al., 2012; Taghizadeh et al., 2020). It will be upon future studies to determine how such inter-arial coherence is altered by training to perform cognitive tasks.

## STAR★Methods

### Key resources table

**Table:** 

REAGENT or RESOURCE	SOURCE	IDENTIFIER
**Deposited data**		

Figure Data	This paper	https://doi.org/10.17632/z3n7vjh2cz.1

**Experimental models: Organisms/strains**		

Rhesus macaques (*Macaca mulatta*)	Alpha Genesis	N/A

**Software and algorithms**		

MATLAB	MathWorks	R2015-2022a
Chronux	http://chronux.org/	v2.12
FieldTrip	http://www.fieldtriptoolbox.org/	Fieldtrip-20190911

**Other**		

Microelectrodes	FHC	UEWLGGSE4N1E

### Resource availability

#### Lead contact

Further information and requests for resources and reagents should be directed to and will be fulfilled by the lead contact, Dr. Christos Constantinidis (Christos.Constantinidis.1@vanderbilt.edu).

#### Materials availability

This study did not generate new unique reagents.

### Experimental model and subject details

Data were analyzed from two male rhesus monkeys (*Macaca mulatta*), ages 5–9 years old, weighing 5–12 kg. None of these animals had any prior experimentation experience at the onset of our study. Monkeys were either single-housed or pair-housed in communal rooms with sensory interactions with other monkeys. All experimental procedures followed guidelines set by the U.S. Public Health Service Policy on Humane Care and Use of Laboratory Animals and the National Research Council’s Guide for the Care and Use of Laboratory Animals and were reviewed and approved by the Wake Forest University Institutional Animal Care and Use Committee under protocol number A14-196.

### Method details

Monkeys sat with their heads fixed in a primate chair while viewing a monitor positioned 68 cm away from their eyes with dim ambient illumination and were required to fixate on a 0.2° white square appearing in the center of the screen. During each trial, the animals were required to maintain fixation on a 0.2° white square appearing in the center of the screen while visual stimuli were presented either at a peripheral location or over the fovea, in order to receive a liquid reward (typically fruit juice). Any fixation break immediately terminated the trial and no reward was given. Eye position was monitored throughout the trial using a non-invasive, infrared eye position scanning system (model RK-716; ISCAN, Burlington, MA). The system achieved a <0.3° resolution around the center of vision. Eye position was sampled at 240 Hz, digitized and recorded. The visual stimulus display, monitoring of eye position, and synchronization of stimuli with neurophysiological data was performed with in-house software implemented on the MATLAB environment (Mathworks, Natick, MA), utilizing the Psychophysics Toolbox (Meyer and Constantinidis, 2005).

#### Behavioral task

##### Pretraining task

Following a brief period of fixation training and acclimation to the stimuli, monkeys were required to fixate on a center position while stimuli were displayed on the screen. The stimuli shown in the pre-training, passive, spatial task consisted of white 2° squares, presented in one of nine possible locations arranged in a 3 × 3 grid with 10° of distance between adjacent stimuli. The stimuli shown in the pre-training passive feature task consisted of white 2° geometric shapes drawn from a set comprising a circle, diamond, the letter H, the hashtag symbol, the plus sign, a square, a triangle, and an inverted Y-letter. These stimuli could also be presented in one of nine possible locations arranged in a 3 × 3 grid with 10° distance between adjacent stimuli.

Presentation began with a fixation interval of 1 s where only the fixation point was displayed, followed by 500 ms of stimulus presentation (referred to hereafter as cue), followed by a 1.5 s “delay” interval where, again, only the fixation point was displayed. A second stimulus was subsequently shown for 500 ms. In the spatial task, this second stimulus would be either identical in location to the initial stimulus, or diametrically opposite the first stimulus. In the feature task, this second stimulus would appear in the same location to the initial stimulus and would either be an identical shape or the corresponding non-match shape (each shape was paired with one non-match shape). Only one nonmatch stimulus was paired with each cue, so that the number of match and nonmatch trials were balanced in each set. In both the spatial and feature task, this second stimulus display was followed by another “delay” period of 1.5 s where only the fixation point was displayed. The location and identity of stimuli was of no behavioral relevance to the monkeys during the pre-training phase, as fixation was the only necessary action for obtaining reward.

##### Post-training task

The monkeys were then trained to perform working memory tasks that involved the presentation of identical stimuli as the spatial and feature tasks during the pre-training phase. Now monkeys were required to remember the spatial location and/or shape of the first presented stimulus, and report whether the second stimulus was identical to the first or not, via saccading to one of two target stimuli (green for matching stimuli, blue for non-matching). Each target stimulus could appear at one of two locations orthogonal to the cue/sample stimuli, pseudo-randomized in each trial.

#### Surgery and neurophysiology

A 20 mm diameter craniotomy was performed over the PFC and a recording cylinder was implanted over the site. The location of the cylinder was visualized through anatomical magnetic resonance imaging (MRI) and stereotaxic coordinates post-surgery. Electrode penetrations were mapped onto the cortical surface. We identified 6 lateral PFC regions: a posterior-dorsal region that included area 8A, a mid-dorsal region that included area 8B and area 9/46, an anterior-dorsal region that included area 9 and area 46, a posterior-ventral region that included area 45, an anterior-ventral region that included area 47/12, and a frontopolar region that included area 10 (Riley et al., 2017). Only posterior dorsal, mid-dorsal and posterior-ventral areas were sufficiently sampled and were included in these analyses.

#### LFP recordings

Recordings were carried out in the aforementioned areas of the PFC both before and after training in each WM task. Extracellular recordings were performed with multiple microelectrodes that were either glass- or epoxylite-coated tungsten, with a 250 μm diameter and 1–4 MΩ impedance at 1 kHz (Alpha-Omega Engineering, Nazareth, Israel). A Microdrive system (EPS drive, Alpha- Omega Engineering) advanced arrays of up to 8-microelectrodes, spaced 0.2–1.5 mm apart, through the dura and into the PFC. The signal from each electrode was amplified and band-pass filtered between 0.5 and 200 Hz for LFP signals and between 500 Hz and 8 kHz for spiking activity (not included in this analysis) with a modular data acquisition system (APM system, FHC, Bowdoin, ME). Identical data collection procedures, recording equipment, and spike sorting algorithms were used before and after training in order to prevent any analytical confounds.

### Quantification and statistical analysis

We used the FieldTrip toolbox (Oostenveld et al., 2011) for preprocessing analysis and Chronux package (Bokil et al., 2010) for time-frequency analysis. For power analysis of LFP signals from the recording electrodes, a band-pass filter (0.5–200 Hz) was used. We removed line power (60 Hz) and other artifacts from each electrode and trial in the LFP signal, if present. We used a multi-taper method to perform a power spectrum analysis of LFP. The spectrogram of each single trial between 0.5 and 100 Hz were computed with 11 tapers of 500 msec time windows with steps of 100 msec. LFP power can be calculated in two ways, referred to as induced and evoked power, respectively. To calculate induced power, the power computation was performed first in each trial. Then power across trials was averaged. Induced power thus determines power at specific frequencies that may not be synchronized with specific task events across trials. To calculate evoked power, raw voltage signals from one site were averaged first, and then power was computed based on this averaged signal. Evoked power thus emphasizes power synchronized at specific task events, common across trials; unless power elevation is synchronized in such a fashion, periodic signals of different phases in different trials will tend to cancel each other when averaged together prior to power computation. Both induced and evoked power in our analysis, was expressed relative to the mean power recorded during the inter-trial interval, which included both task events. Time-resolved plots (spectrograms) were constructed and plotted after dividing the power of the signal by the mean inter-trial interval power at each frequency (which is equivalent to subtracting the baseline power in logarithmic, dB, scale).

Statistical testing of differences between conditions was performed in the following fashion. First we calculated power across an entire epoch: fixation period, cue presentation, first delay, sample presentation, second delay (rather than at every time point, as illustrated in spectrograms). Secondly, we averaged power values in these epochs from all trials of every electrode site, essentially treating each LFP site as one observation. We then constructed a 3-way ANOVA model, with factors pre- or post-training condition; prefrontal subdivision; and task epoch. We repeated this analysis for each of four frequency bands defined as alpha (8–14 Hz), beta (16–32 Hz), gamma (33–64 Hz) and high-gamma (65–100 Hz).

## Data Availability

• Data used for the analysis and figures will be deposited at Mendeley.com and made publicly available as of the date of publication. DOIs are listed in the key resources table. • This paper does not report original code • Any additional information required to reanalyze the data reported in this paper is available from the lead contact upon request.
