# Supplementary material for: Plasticity after cognitive training reflected in prefrontal local field potentials
Source: iScience. 2022 Aug 12;25(9):104929. doi: 10.1016/j.isci.2022.104929 (PMC9440296; doi:10.1016/j.isci.2022.104929)
Supplement: Document S1. Figures S1–S4 [file mmc1.pdf]

iScience, Volume *25*

Supplemental information

Plasticity after cognitive training reflected in prefrontal local field potentials

Balbir Singh, Zhengyang Wang, Xue-Lian Qi, and Christos Constantinidis

Inventory of Supplemental Information

Figure S1. Induced LFP power in two hemispheres, related to Figure 2

Figure S2. Time course of power in the feature task, related to Figure 2.

Figure S3. Induced LFP power in two hemispheres for the feature task, related to Figure 5.

Figure S4. Time course of power in each area post-training, related to Figure 3.

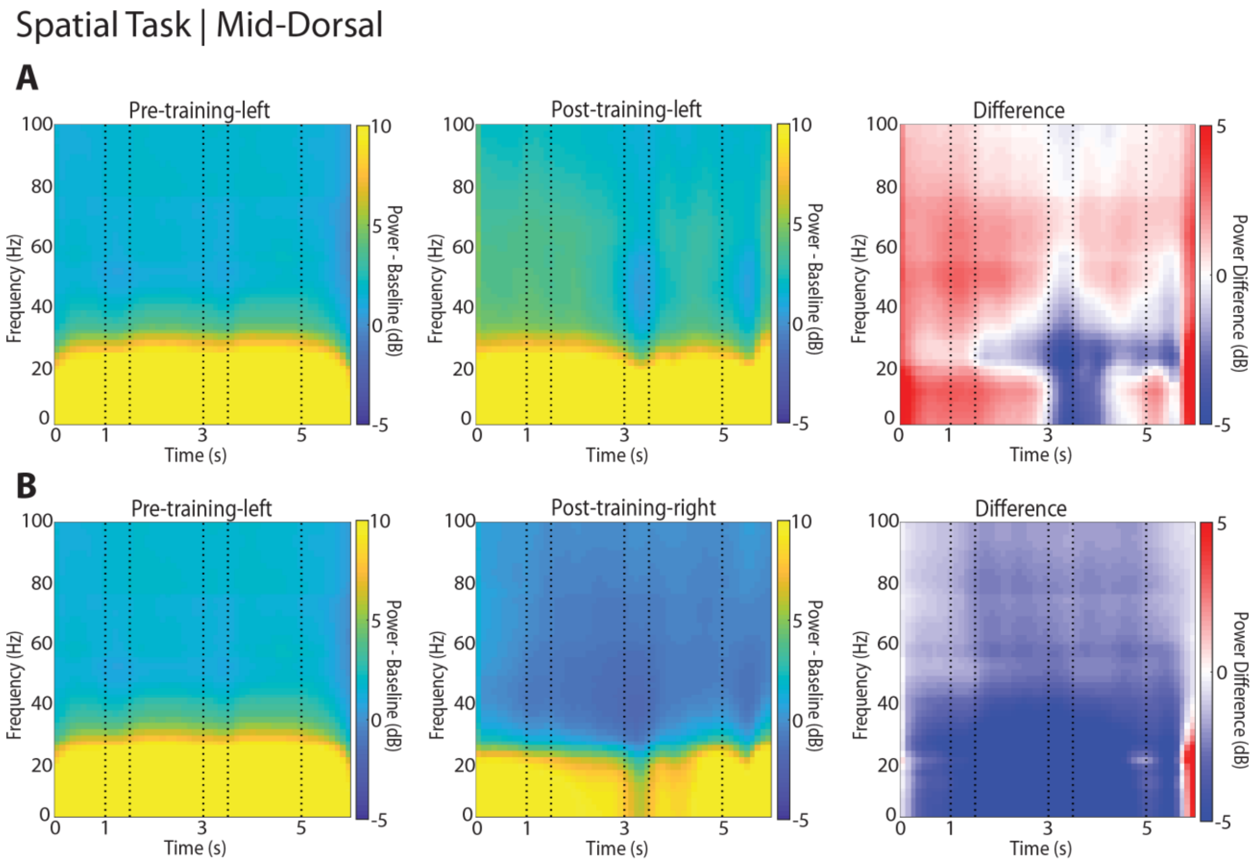

Supplemental Figure S1. Induced LFP power in two hemispheres, related to Figure 2. LFP spectral power recorded with the spatial set from the mid-dorsal region of the prefrontal cortex, prior to training (left column) and after training (middle column), as well as their difference (right column). Power is plotted as a function of time, after subtracting the mean power computed in the inter-trial interval at each frequency band. Horizontal lines indicate time of the two stimulus presentations (at 1-1.5 and 3-3.5 s). Results are shown (A) for the left hemisphere before and after training in the spatial task (n=13811, and 5917). (B) From the right hemisphere after training in the spatial task (n=9646). Left plot is the same in panels A and B.

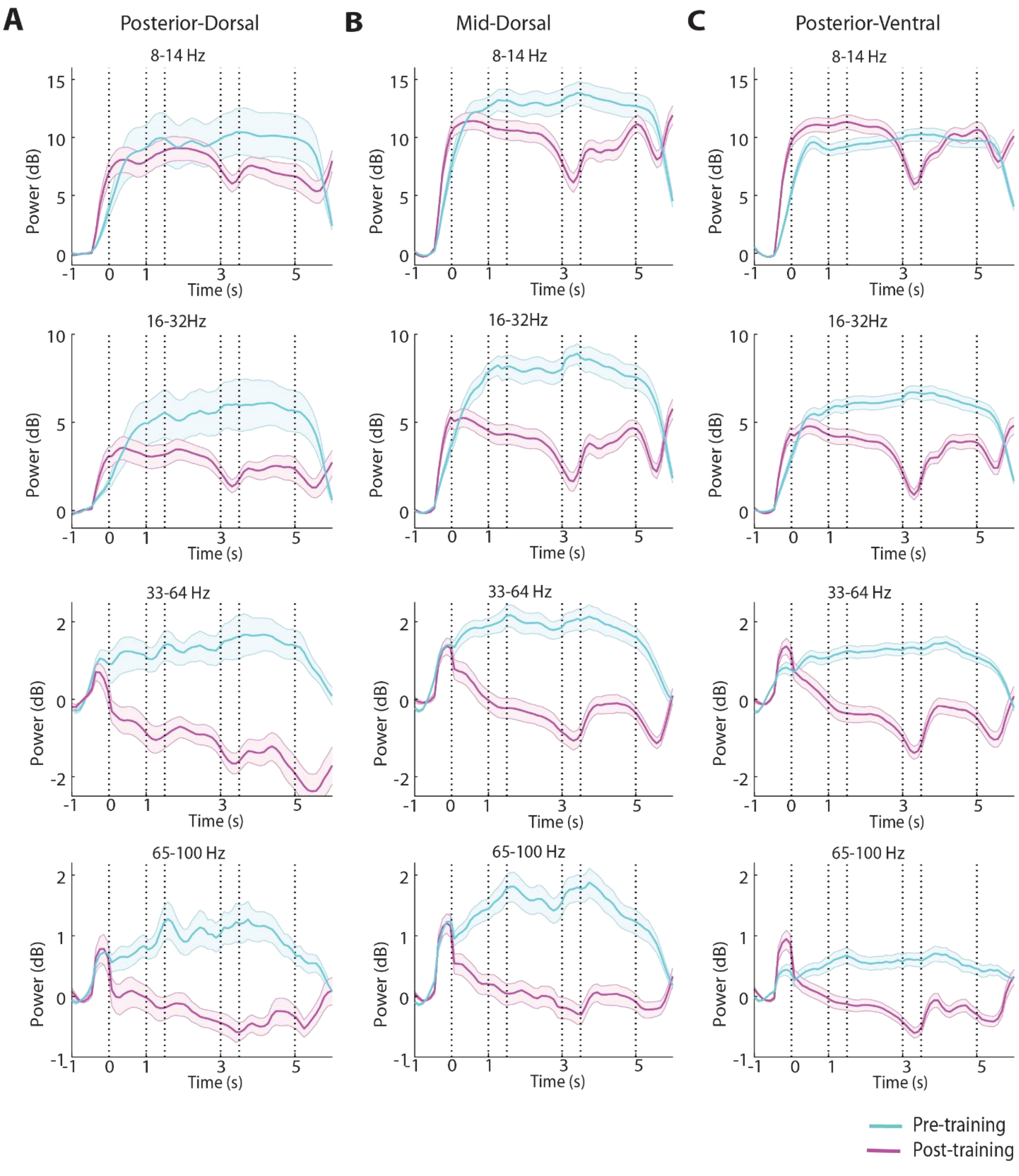

Supplemental Figure S2. Time course of power in the feature task related to Figure 2. Time course of induced spectral power in the feature task, after subtracting the mean, in the alpha (8-14), beta (16-32), gamma (33-64) and high-gamma (65-100) frequency band, comparing pre-training and post-training results. Results are shown separately for (A) posterior-dorsal (n=15 and 37 sites for pre-training and post-training, respectively), (B) mid-dorsal (n=47 and 57) and (C) posterior-ventral areas (n=105 and 121). Data are represented as mean (solid line) and its 5 and 95 percentiles estimated by subsampling 75% of the data 1000 times (shaded area).

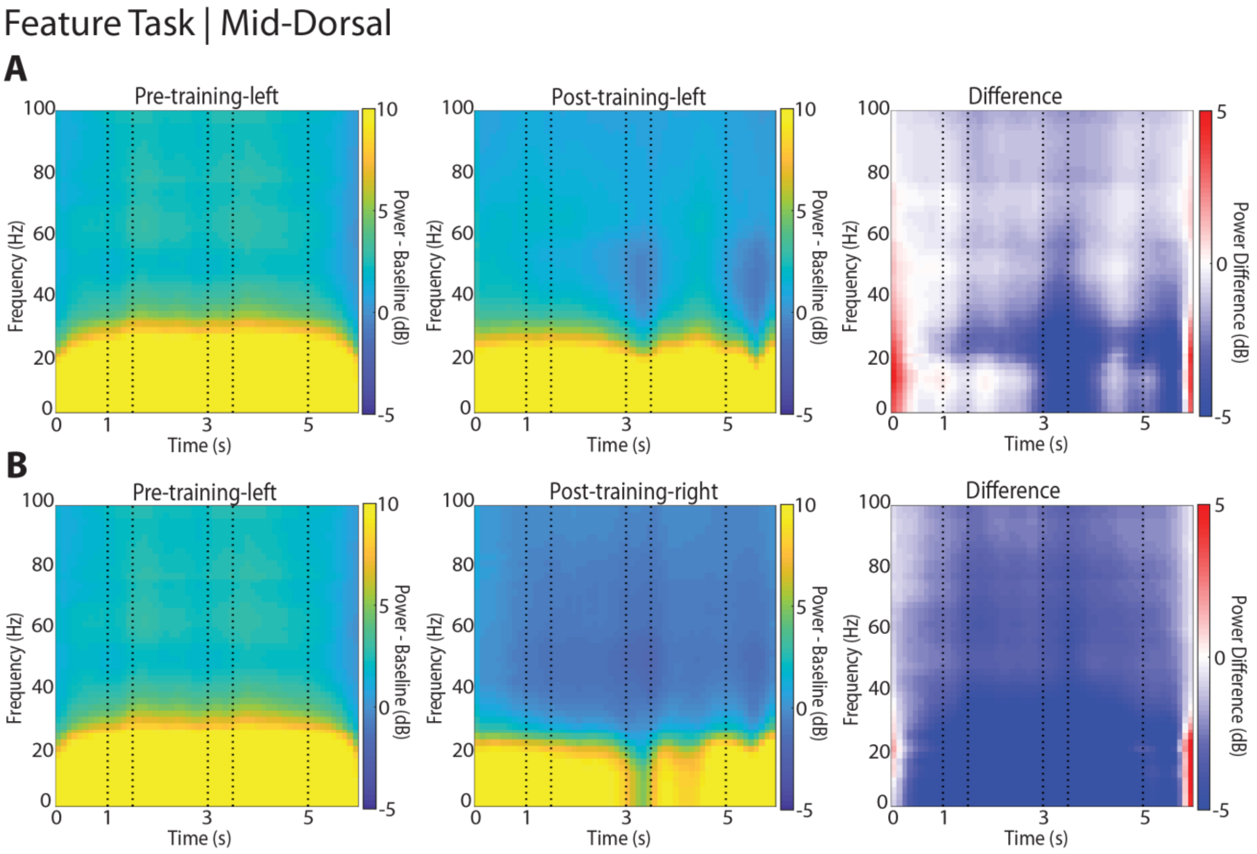

Supplemental Figure S3. Induced LFP power in two hemispheres for the feature task, related to Figure 5. LFP spectral power recorded with the feature stimuli from the mid-dorsal region of the prefrontal cortex during the time course of the trial, prior to training (left column) and after training (middle column), as well as their difference (right column). Power is plotted as a function of time, after subtracting the mean power computed in the inter-trial interval at each frequency band. Horizontal lines indicate time of the two stimulus presentations (at 1-1.5 and 3-3.5 s). (A) Results from the mid-dorsal area of the left hemisphere before and after training in the feature task (n=4395 and 1953 trials, respectively). (B) Results from the mid-dorsal area of the right hemisphere after training (n=3886). Left plot is the same in panels A and B.

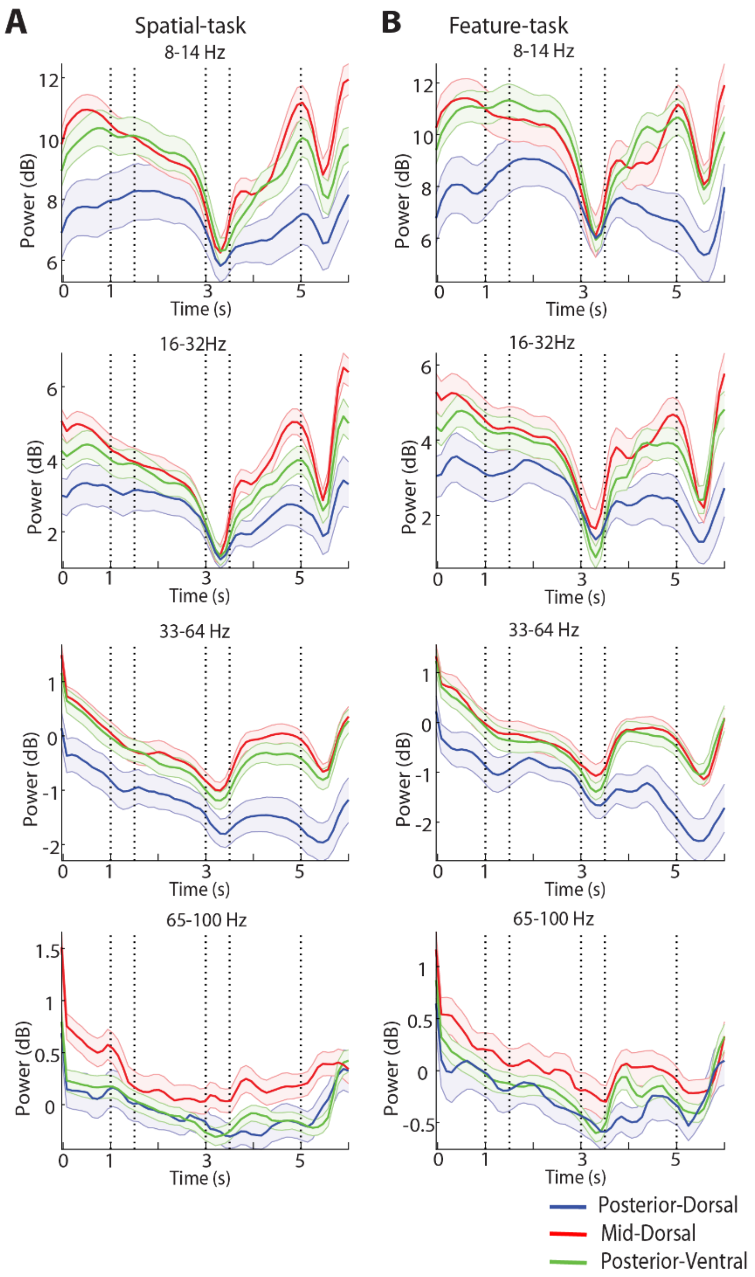

Supplemental Figure S4. Time course of power in each area post-training, related to Figure 3. Time course of induced spectral power in the spatial and feature tasks after training, in the alpha (8-14), beta (16-32), gamma (33-64) and high-gamma (65-100) frequency band. The same datasets are shown as in Figure 3 and S2, now organized by area.
